# Profil épidémio-clinique et évolutif des cardiomyopathies dilatées au Centre Hospitalier Universitaire de Brazzaville, Congo

**DOI:** 10.11604/pamj.2018.31.164.16477

**Published:** 2018-11-07

**Authors:** Stéphane Méo Ikama, Bijou Moualengue, Jospin Makani, Solange Flore Mongo-Ngamami, Bertrand Ellenga-Mbolla, Igor Ondze-Kafata, Christian Kouala-Landa, Thierry Raoul Gombet, Gisèle Kimbally Kaky

**Affiliations:** 1 Service de Cardiologie et Médecine Interne, Centre Hospitalier Universitaire de Brazzaville, Congo; 2 Faculté des Sciences de la Santé, Université Marien Ngouabi de Brazzaville, Congo

**Keywords:** Cardiomyopathie dilatée, fréquence, pronostic, Congo, Dilated cardiomyopathies, frequency, prognosis, Congo

## Abstract

L'objectif de cette étude est de contribuer à l'amélioration de la prise en charge des patients porteurs de cardiomyopathies dilatées (CMD) à Brazzaville. Cette étude, prospective et analytique, a été réalisée au CHU de Brazzaville entre le 1^er^ Janvier 2014 et le 30 Juin 2015. Elle a inclus les patients hospitalisés dans le service de cardiologie pour une insuffisance cardiaque (IC) en rapport avec une CMD. L'étude a porté sur 100 patients. La fréquence hospitalière de la CMD était de 32,1%. Il s'agissait de 38 hommes (38%) et 62 femmes (62%), âgés en moyenne de 52,9 ± 17,1 ans. L'IC était globale dans 72 cas (72%). L'ECG s'inscrivait en rythme sinusal (95%), et objectivait une hypertrophie ventriculaire gauche (40%), un bloc de branche gauche (16%), et une fibrillation auriculaire (5%). La fraction d'éjection du ventricule gauche (VG) était en moyenne de 33,4 ± 6,8%, et le diamètre télédiastolique du VG de 65,5 ± 7,0 mm. Le traitement comportait un diurétique de l'anse (100%), un IEC/ARA2 (100%), un bêtabloquant (38%), un digitalique (30%), un anti-aldostérone (16%), et un anti-vitamine K (11%). Au terme d'un suivi de 12 mois, le taux de létalité globale était de 9%, le taux de réhospitalisation de 12%, et le taux de perdus de vue de 41%. Cette étude a montré que la CMD est une affection fréquente, et une des principales causes d'insuffisance cardiaque. La durée de suivi brève et le nombre important de perdus de vue ne permettent pas d'en évaluer la survie dans notre contexte.

## Abstract (en)

This study aims to contribute to the improvement of treatment protocols for patients with dilated cardiomyopathies (DCMs) in Brazzaville. We conducted a prospective analytical study at the University Hospital in Brazzaville between 1 January 2014 and 30 June 2015. All patients hospitalized with heart failure (HF) associated with DCM in the Department of Cardiology were included in the study. The study involved 100 patients. Hospitalization rate for DCM was 32.1%: 38 men (38%) and 62 women (62%) with an average age of 52.9 ± 17.1 years. Seventy two patients had comprehensive heart failure (72%). ECG showing normal sinus rhythm (95%) objectified left ventricular hypertrophy (40%), left bundle-branch block (16%), atrial fibrillation (5%). Mean left ventricular ejection fraction (EF) was 33.4 ± 6.8% and left ventricle end-diastolic diameter was 65.5 ± 7.0 mm. Treatment was based on loop diuretic (100%), ACE Inhibitors, Angiotensin II Receptor Blockers (ARBs) (100%), beta blocker (38%), digitalis (30%), anti-aldosterone (16%) and anti-vitamin K (11%). After 12-month follow-up period, overall case-fatality rate was 9%, readmission rate was 12% and the rate of patient lost-to-follow-up was 41%. This study shows that DCM is frequent and it is one of the leading causes of heart failure. The short follow-up period and the high rate of people lost to follow up do not enable assessment of survival rate of patients at our Department.

## Introduction

La cardiomyopathie dilatée (CMD), définie par la dilatation du ventricule gauche ou des deux ventricules, associée à une altération de la fonction systolique du ventricule gauche, est la plus fréquente des cardiomyopathies, et constitue la principale cause d'insuffisance cardiaque (IC) dans le monde [1-4]. Il s'agit d'une affection grave, de mauvais pronostic en raison de la forte mortalité qui lui est imputable, de l'ordre de 15 à 50% [3]. Cependant, les progrès thérapeutiques notés au cours de ces dernières décennies, notamment avec l'utilisation des bêtabloquants, ont permis une amélioration considérable de la survie à moyen et long terme des patients [5, 6]. Au Congo, la CMD représente la deuxième cause d'IC après l'hypertension artérielle, et pose le problème des difficultés dans la prise en charge en raison de la précarité des populations [7]. La présente étude, visant à améliorer la prise la prise en charge des patients insuffisants cardiaques en rapport avec une CMD, s'est fixée pour objectifs de déterminer la fréquence de cette affection au sein des maladies cardiovasculaires, et d'en évaluer le devenir des patients dans notre contexte.

## Méthodes

Il s'est agi d'une étude longitudinale à recueil de données prospectif, analytique et comparatif, qui s'est déroulée dans le service de cardiologie et médecine interne du CHU de Brazzaville, sur une période de suivi de 18 mois, allant du 1^er^ janvier 2014 au 30 juin 2015. Elle a inclus les patients hospitalisés pour insuffisance cardiaque gauche ou globale en rapport avec une CMD (définie par une dilatation du ventricule gauche > 55 mm ou 27 mm/m^2^ de diamètre télédiastolique, ou des deux ventricules, associée à une baisse de la fraction d'éjection < 45%), et présentant un dossier médical complet comportant une radiographie thoracique, un électrocardiogramme (ECG), une échocardiographie, et un bilan biologique (hémogramme, créatininémie avec débit de filtration glomérulaire, ionogramme sanguin, vitesse de sédimentation, CRP et sérologie HIV le cas échéant). N'ont pas été inclus dans l'étude, les patients présentant une IC en rapport avec une cause autre que la CMD, les patients porteurs de CMD décédés en cours d'hospitalisation, et les patients sortis contre avis médical. Les variables d'étude ont été:

**Sociodémographiques**: fréquence, âge, sexe, provenance, niveau d'instruction (aucun, primaire, secondaire, supérieur), niveau de vie (faible, moyen, élevé) selon ECOM [8].

**Cliniques**: type d'IC (IC gauche ou globale), l'ancienneté, les hospitalisations antérieures et les facteurs de décompensation.

**Paracliniques**: radiologiques (index cardio-thoracique), électrocardiographiques (rythme sinusal ou ACFA, trouble d'excitabilité, bloc de branche ou bloc auriculo-ventriculaire, repolarisation et onde Q de nécrose), échocardiographiques (diamètres ventriculaires, taille des oreillettes, FEVG, pressions pulmonaires, état du péricarde, thrombus intra-cavitaire le cas échéant) et biologiques.

**Thérapeutiques**: traitements prescrits (à visée anti-insuffisance cardiaque et à visée anti-thrombotique) et l'observance médicamenteuse (bon observant, non-observant mineur et non observant défini selon le test mis au point et validé par Girerd *et al*. par un total des oui égal à 0, compris entre 1-2 et ≥ 3 respectivement) [9].

**Evolutives**: durée du séjour, IC compensée ou non, réhospitalisations, décès.

Pour le suivi des patients, une réévaluation a été faite à 6 mois et à 12 mois sur la base des données cliniques, paracliniques et thérapeutiques. Pour la taille de l'échantillon, nous avons procédé par un échantillonnage de convenance. Sur les 536 patients hospitalisés pour IC durant la période d'étude, 172 présentaient une CMD. Pour l'étude, seuls 100 cas de CMD répondant aux critères d'inclusion ont été retenus. Les données ont été traitées et analysées avec le logiciel Epi-info 3.5.1. Les tests de Khi-2 et ANOVA ont été utilisés pour la comparaison des variables qualitatives et quantitatives. Le seuil de significativité a été fixé à p < 0,05. Pour les considérations éthiques, un avis favorable a été obtenu auprès du Comité d'éthique de la recherche en sciences de la santé (CERSSA).

## Résultats

**Figure 1:**
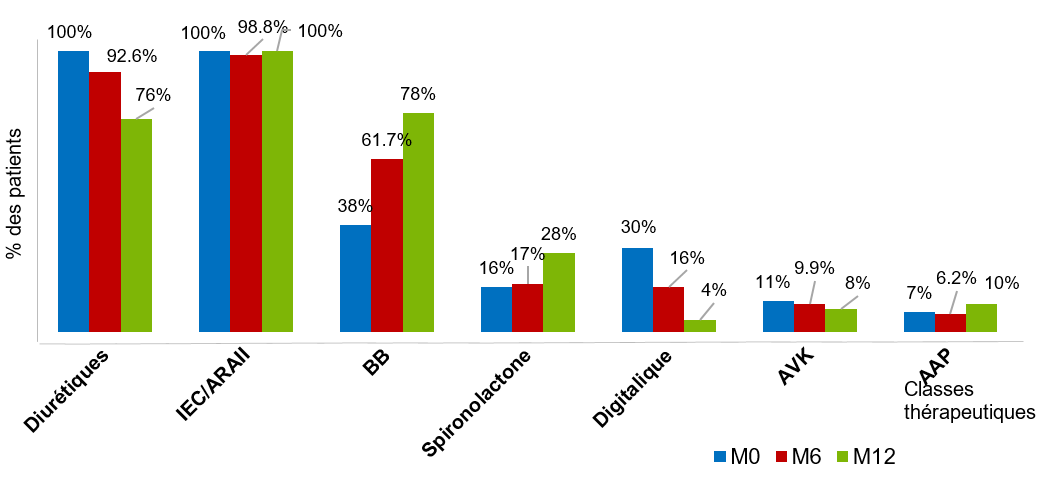
Évolution des prescriptions médicamenteuses au cours du suivi

**Figure 2:**
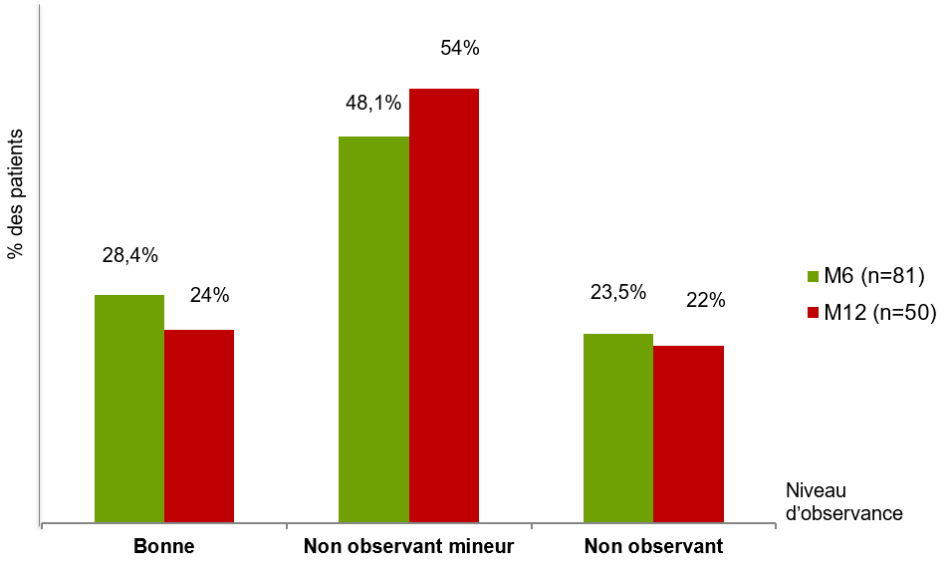
Évolution des niveaux d’observance médicamenteuse au cours du suivi

La CMD représentait 26% de l'ensemble des admissions pour affections cardiovasculaires et la deuxième cause d'hospitalisation pour insuffisance cardiaque après l'hypertension artérielle, avec une fréquence de 32,1%. Les 100 patients inclus se répartissaient en 38 hommes (38%) et 62 femmes (62%). L'âge moyen des patients était de 52,9 ± 17,1 ans (extrêmes: 15 et 86 ans), dont 50% âgés de moins de 55 ans. Les patients avaient un niveau d'instruction primaire dans 17 cas (17%), secondaire dans 49 cas (49%), et supérieur dans 9 cas (9%). Ils étaient d'un niveau de vie faible dans 43 cas (43%), moyen dans 22 cas (22%), et élevé dans 35 cas (35%). L'insuffisance cardiaque était globale dans 72 cas (72%), et les patients en classe fonctionnelle NYHA III-IV dans 75 cas (75%). La cardiomégalie était constante avec un rapport cardio-thoracique moyen de 63,2 ± 6,4% (extrêmes: 56 et 83%). L'ECG s'inscrivait en rythme sinusal dans 95 cas (95%), et objectivait une fibrillation auriculaire dans 5 cas (5%), une hypertrophie ventriculaire gauche dans 40 cas (40%), un bloc de branche gauche dans 16 cas (16%). L'échocardiographie transthoracique objectivait une hypokinésie globale et une dysfonction systolique dans tous les cas, avec une fraction d'éjection du ventricule gauche (FEVG) de 33,4 ± 6,8% en moyenne (extrêmes: 17 et 45%). Le diamètre télédiastolique du ventricule gauche (DTDVG) était en moyenne de 65,5 ± 7,0 mm (extrêmes: 56 et 90 mm), et celui du ventricule droit de 26,7 ± 6,3 mm (extrêmes: 17 et 43 mm). L'oreillette gauche était dilatée dans 79 cas (79%) avec une surface moyenne de 26,5-7,1 cm^2^ (extrêmes: 12 et 48 cm^2^), et l'oreillette droite dans 40 cas (40%) avec une surface moyenne de 19,3+/-6,4 cm^2^ (extrêmes: 10 et 38 cm^2^). Une hypertension artérielle pulmonaire (HTAP) était observée dans 33 cas (33%), avec une PAPs moyenne de 29,8 ± 16,9 mmHg. Un thrombus a été retrouvé à l'apex du VG dans un cas, et un épanchement péricardique minime dans 13 cas (13%). Le traitement de sortie comportait un diurétique de l'anse dans tous les cas (100%), un IEC/ARA2 dans tous les cas (100%), un bêtabloquant dans 38 cas (38%), un digitalique dans 30 cas (30%), un antagoniste des récepteurs des minéralocorticoïdes (spironolactone) dans 16 cas (16%), un anti-vitamine K dans 11 cas (11%), et un antiagrégant plaquettaire (aspirine) dans 7 cas (7%). La durée moyenne de séjour a été de 32,4+/-15,4 jours (extrêmes: 15 et 45 jours). Les principales caractéristiques de la population d'étude sont représentées dans le Tableau 1. L'évolution au terme de six mois de suivi, permettait de noter huit cas de décès (8%), 11 perdus de vue (11%), et sept réhospitalisations pour IC soit 8,6% des 81 patients encore présents. Au terme des douze mois de suivi, plus que 50 patients étaient présents, avec un cas de décès, cinq cas de réhospitalisations et 30 perdus de vue. Le taux de létalité globale au cours du suivi était de 9%, le taux de réhospitalisations pour IC de 12%, et le nombre de perdus de vue de 41 patients. Les principales causes de décès étaient une mort subite dans (n = 5), une IC réfractaire (n = 3), et un accident vasculaire cérébral (n = 1). Les facteurs de décompensation identifiés étaient un écart de régime avec arrêt du traitement d'entretien (n = 10), un syndrome grippal (n = 2), et un passage en fibrillation auriculaire (n = 1). Les patients décédés n'étaient pas traités par bêtabloquants dans tous les cas, et les réhospitalisés dans neuf cas sur 12. Les Figure 1 et Figure 2 représentent l'évolution des prescriptions médicamenteuses, et les niveaux d'observance au cours du suivi respectivement. Les Tableau 2 et Tableau 3 montrent l'évolution des paramètres échocardiographiques au cours du suivi, et l'impact des bêtabloquants sur la FEVG respectivement.

**Tableau 1:** Principales caractéristiques des patients

Variables	Patients (N = 100)
Femmes, n (%)	62 (62)
Age moyen (ans)	52,9 ± 17,1 (15 - 86)
Niveau d’instruction secondaire, n (%)	49 (49)
Secteur informel, n (%)	39 (39)
Revenu moyen (Fcfa)	80.000 ± 81.639
IC globale, n(%)	72 (72)
Fibrillation atriale, n (%)	5 (5)
DTDVG moyen (mm)	65,5 ± 7,0 (56 - 90)
DTDVD moyen (mm)	26,7 ± 6,3 (13 - 43)
FEVG moyenne (%)	33,4 ± 6,8 (17 et 45%)
Diurétiques, n (%)	100 (100)
IEC/ARA2, n (%)	100 (100)
Bétabloquants, n (%)	38 (38)
Spironolactone, n (%)	16 (16)
Digoxine, n (%)	30 (30)

**ARA2**: antagoniste des récepteurs de l’angiotensine II; **DTDVG**: diamètre télédiastolique du ventricule gauche; **DTDVD**: diamètre télédiastolique du ventricule droit; **FEVG**: fraction d’éjection du ventricule gauche; **IC**: insuffisance cardiaque; **IEC**: inhibiteur de l’enzyme de conversion

**Tableau 2:** Évolution des paramètres échocardiographiques au cours du suivi

	MO	M6	M12	P
DTDVG (mm)	65,5 ± 7	62,1 ± 9,2	59,8 ± 9,6	0,0001
DTDVD (mm)	26,8 ± 6,4	25,6 ± 4,7	26,2 ± 4,4	0,5
FEVG (%)	33,4 ± 6,8	43,9 ± 12,1	47,0 ± 1,3	0,0001

**DTDVG:** diamètre télédiastolique du ventricule gauche; **DTDVD:** diamètre télédiastoilique du ventricule droit; **FEVG:** fraction d’éjection du ventricule gauche; **M0:** à l’inclusion; **M6:**au sixième mois; **M12:** au douzième mois

**Tableau 3:** Impact des bétabloquants sur la fraction d’éjection du ventricule gauche (FEVG)

	FEVG et bétabloquants		
	Oui	Non	p
M0	33,8 ± 7,3%	33,2 ± 6,6%	0,64
M6	46,7 ± 10,9%	39,8 ± 13,2%	0,02
M12	48,6 ± 13%	41,2 ± 13%	0,01

## Discussion

En Afrique subsaharienne, sur des données essentiellement hospitalières, la cardiomyopathie dilatée (CMD) constitue l'une des principales causes d'insuffisance cardiaque (IC), avec une fréquence hospitalière variant entre 17 et 48% [10-11]. Dans notre série, la CMD représentait la deuxième affection cardiovasculaire, et la deuxième cause d'insuffisance cardiaque dans près d'un-tiers des cas, après la cardiopathie hypertensive. En Europe, la CMD est responsable de 20-50% des hospitalisations pour IC après la cardiopathie ischémique [12, 13]. Dans notre étude, comme dans d'autres séries africaines, les patients sont relativement jeunes, avec un âge moyen autour de 50 ans [14-16], comparativement aux séries occidentales où les patients sont souvent plus âgés, en raison de l'espérance de vie plus longue dans cette partie du monde [17]. La prédominance masculine, de règle dans cette affection, touchant près de trois hommes pour une femme [3, 13, 18, 19], n'a pas été retrouvée dans notre série. L'insuffisance cardiaque est la principale expression clinique de la CMD dans notre série. En effet, l'IC constitue la principale circonstance de découverte de la CMD retrouvée dans près de 8 cas sur dix, loin devant d'autres circonstances moins fréquentes telles qu'une douleur thoracique pseudo-angineuse, des palpitations en rapport avec un trouble du rythme, des manifestations thrombo-emboliques, ou plus rarement de manière fortuite [1, 18]. Cette IC est le plus souvent sévère dans notre série, attestée par la prépondérance de la classe fonctionnelle NYHA III-IV, l'importance de la dilatation des cavités cardiaques, et le degré d'altération de la fonction systolique du ventricule gauche.

Au cours du suivi, une amélioration clinique a été notée chez plus de huit patients sur dix au sixième mois, marquée par une stabilité hémodynamique avec résolution quasi-complète des signes d'insuffisance cardiaque. Cette évolution favorable s'est traduite également sur le plan échocardiographique par l'amélioration aussi bien des dimensions des cavités cardiaques, que de la fonction systolique du ventricule gauche (FEVG). En effet, il a été constaté une amélioration significative de la FEVG au cours du suivi, au sixième et au douzième mois, avec un bénéfice considérable chez les patients traités par bétabloquants, comparativement aux patients qui n'étaient pas sous bétabloquants. Ce bénéfice apporté par les bétabloquants s'est traduit en termes de diminution de la mortalité et du nombre de réhospitalisations. Plusieurs grandes études menées au cours de ces dernières décennies, ont mis en exergue l'efficacité des bétabloquants dans la réduction de la morbi-mortalité chez des patients porteurs de CMD, faisant de cette classe thérapeutique un impératif dans la prise en charge de cette entité nosologique [3, 6, 18, 20]. Dans notre étude, cette classe thérapeutique a été prescrite chez plus du tiers des patients à la sortie du séjour hospitalier, et a vu sa prescription augmentée au cours du suivi, rendant notre attitude conforme à celle recommandée dans la littérature [3, 6, 20]. Dans notre série, le taux de réhospitalisation à 12 mois était de 12%, avec comme principaux facteurs à l'origine de la décompensation, les écarts de régime et l'arrêt du traitement d'entretien, le tout sur fond d'inobservance thérapeutique, notée chez près de 3/4 des patients. Le bas niveau socio-économique des patients, l'absence de la couverture maladie universelle, et le manque d'éducation thérapeutique des patients peuvent être l'explication [6, 21, 22]. La mortalité globale était de l'ordre de 9% à 12 mois dans notre série. Ce taux de mortalité est probablement sous-estimé du fait non seulement de la courte période de suivi, mais aussi et surtout du nombre important de patients perdus de vue, la mort subite étant une modalité évolutive fréquente au cours de cette affection. D'autres auteurs africains ont rapporté des résultats similaires [14]; mais globalement, d'après les données de la littérature, la mortalité à 5 ans associée aux CMD varie de 15 à 50% [1, 18, 23]. Les principales causes de décès au cours de cette affection sont représentées, à des degrés divers selon les auteurs, par la mort subite, l'insuffisance cardiaque réfractaire, les troubles du rythme, et plus rarement les complications thrombo-emboliques [1, 14, 18, 24].

## Conclusion

Cette étude a montré que la CMD est une affection fréquente, et une des principales causes d'insuffisance cardiaque. Elle est responsable d'une mortalité non négligeable. L'effet bénéfique du traitement par bétabloquants sur le pronostic a été mis en exergue dans cette étude, se traduisant en termes de réduction de la mortalité et du taux de réhospitalisations pour IC. Cependant, la durée de suivi brève et le nombre important de perdus de vue ne permettent pas d'en évaluer la survie dans notre contexte.

### Etat des connaissances actuelles sur le sujet

- La CMD est une cause fréquente d'insuffisance cardiaque;
- Il s'agit d'une affection grave, en raison du risque élevé de mort subite;
- Son pronostic a été amélioré au cours de ces dernières décennies grâce à l'utilisation des bétabloquants et du défibrillateur automatique implantable (DAI).

### Contribution de notre étude à la connaissance

- En raison du changement du cadre nosologique des affections cardiovasculaires dans nos pays, cette étude a permis de repréciser la place de la CMD;
- Si sa prise en charge est bien codifiée en Occident, la présente étude a montré les difficultés de cette prise en charge dans notre contexte, attestées par le nombre considérable de patients perdus de vue, et le faible niveau d'observance médicamenteuse, probablement du fait des conditions de vie précaires;
- Enfin, la nécessité de l'utilisation des bétabloquants a été également mise en exergue dans cette étude en termes d'amélioration du pronostic.

## Conflits des intérêts

Les auteurs ne déclarent aucun conflit d'intérêts.
